# Analyses of the return on investment of public health interventions: a scoping review and recommendations for future studies

**DOI:** 10.1136/bmjgh-2023-012798

**Published:** 2023-08-30

**Authors:** Hugo C Turner, Yoshiaki Hori, Paul Revill, Waranya Rattanavipapong, Ko Arai, Justice Nonvignon, Mark Jit, Yot Teerawattananon

**Affiliations:** 1 MRC Centre for Global Infectious Disease Analysis, School of Public Health, Imperial College London, London, UK; 2 School of Public Health, Imperial College London, London, UK; 3 Centre for Health Economics, University of York, York, UK; 4 Health Intervention and Technology Assessment Program (HITAP), Ministry of Public Health, Nonthaburi, Thailand; 5 Graduate School of Business Administration, Hitotsubashi University, Tokyo, Japan; 6 School of Public Health, University of Ghana, Accra, Ghana; 7 Africa Centres for Disease Control and Prevention, Addis Ababa, Ethiopia; 8 Centre of Global Change and Health, London School of Hygiene & Tropical Medicine, London, UK; 9 School of Public Health, University of Hong Kong, Hong Kong Special Administrative Region, China; 10 Saw Swee Hock School of Public Health, National University of Singapore, Singapore

**Keywords:** health economics, health policy, health policies and all other topics, public health

## Abstract

Return on investment (ROI) analysis is increasingly being used for evaluating the value for money of public health interventions. Given its potential role for informing health policies, it is important that there is a more comprehensive understanding of ROI analysis within the global health field. To address this gap in the literature, we conducted a scoping review of recent research articles reporting an ROI metric for a health intervention within the public sector in any country setting. The database search was limited to literature published in English and studies published between 1 January 2018 and 14 June 2021. Uses and settings where the ROI metric is being applied, key methodological features of the calculations and the types of economic benefits included were extracted. 118 relevant studies were included within this scoping review. We found that ROI analyses of health interventions differed between those that only included fiscal savings (such as prevented medical expenses) and those which incorporated a wider range of benefits (such as monetised health benefits). This highlights the variation in the definition of ROI analyses and supports the finding that ROI analyses are used for a range of different research questions/purposes within the healthcare sector. We also found that the methodologies used in ROI calculations were inconsistent and often poorly reported. This review demonstrates that there is notable variation in the methodology surrounding recent ROI calculations of healthcare interventions, as well as the definition of ROI analysis. We recommend that ROI metrics should be carefully interpreted before they are used to inform policy decisions regarding the allocation of healthcare resources. To improve the consistency of future studies, we also set out recommended use cases for ROI analysis and a reporting checklist.

**WHAT IS ALREADY KNOWN ON THIS TOPIC**

- Although return on investment (ROI) analyses are increasingly being used to evaluate the value for money of health interventions, the details and features of such analysis have not been fully explored in the literature and there is a risk these studies could be misinterpreted.

**WHAT THIS STUDY ADDS**

- This scoping review is the first to comprehensively investigate the uses of ROI analysis within global health, how they are described and their methodology.
- We found that there is notable variation in the methodology surrounding recent ROI analyses of health interventions; such as if the study included only fiscal savings (such as prevented medical expenses) or a wider range of benefits (such as monetised health benefits). This methodological variation is important as it means that studies reporting an ROI are often not directly comparable to one another.

**HOW THIS STUDY MIGHT AFFECT RESEARCH, PRACTICE OR POLICY**

- Based on the variation in its current usage and methodology we recommend a degree of caution using the ROI metric in the context of health technology assessment/priority setting for informing policy decisions surrounding the allocation of healthcare resources. To improve the consistency of future studies, we also set out recommended use cases for ROI analysis and a reporting checklist.

## Introduction

Health economic analyses have an important role in assessing the value for money of health interventions, supporting the optimal allocation of the limited resources available for healthcare.^1 2^ In this context, there are a variety of different types of analysis that can be used to evaluate and compare health interventions (online supplemental box 1); most commonly economic evaluations, such as cost-effectiveness analysis, cost-utility analysis and cost-benefit analysis.^2^ One other metric that is increasingly being used is an intervention’s return on investment (ROI).^3–5^ This is based on looking at the net returns generated by an investment compared with its cost.^4 5^ The following standard formula calculates an ROI^4 5^:

10.1136/bmjgh-2023-012798.supp1 Supplementary data

ROI=(BenefitsorRevenue−Cost)/Cost

Box 1 **Recommendations for future return on investment (ROI) studies**

**Recommended use cases**

We would argue that return of investment analysis can be useful in the following situations;

- The evaluation of fiscal cost savings of interventions. This is useful in situations where the new intervention/strategy is at least as effective as the comparator, particularly in the cases where the health benefits are difficult to quantify into a single measure.
- For an investment case of an intervention or the control of a disease for advocacy purposes.
- When considering the impact of an intervention at a macroeconomic level—such as the impact on gross domestic product.
- For a private company (such as a private insurance company) where the goal is to maximise its revenue.
- The use of social return on investment for evaluating cross-sectoral investments which aim to promote health and development.^16^

In contrast, in situations other than those outlined above we advise caution in using the ROI metric within a health technology assessment/priority setting context when directly comparing the value for money of different interventions to decide which one should be implemented, for which more traditional economic evaluations will typically be more appropriate.

**Recommendations for the reporting of future ROI studies of health interventions**

The following outlines reporting recommendations for ROI studies of health interventions (see online supplemental table S6 for a checklist version). We also recommend that economic evaluations should follow the Consolidated Health Economic Evaluation Reporting Standards (CHEERS) recommendations.^38^ Note that some of these items were adapted from the CHEERS recommendations (indicated with *).

**Introduction**

- Give the context for the study, the study question and its practical relevance for decision-making in policy or practice.*
- Justify the purpose of the analysis, target audience and why ROI is an appropriate metric.

**Methods**

- Clearly describe the following features;*
  - The characteristics of the study population.
  - The interventions or strategies/scenarios being compared and why they were chosen (the comparator or counterfactual).
  - State the perspective(s) adopted by the study and outline why chosen.
  - State the time horizon for the study and outline why it is appropriate.
  - Report the discount rate(s) and outline why chosen.
- Describe the specific ROI calculation being used (ie, how is the ratio or percentage being calculated).
- Report all analytical inputs and parameters (such as values, ranges and references). Include a Table that lists which economic benefits are being included and explicitly how they are being valued monetarily. Clearly stating if the costs relate to fiscal/tangible benefits or not.

**Results**

- Provide a clear breakdown of the ROI stratified by the different types of benefits and stakeholders.
- Report the absolute numbers regarding the cost and benefits and not just the summary ratio/percentage.*
- Report the results stratified by including only fiscal/tangible benefits and non-fiscal.
- If including non-fiscal savings—avoid phrasing such as for every dollar invested generates the ‘US$X’ value in returns.
- Perform a sensitivity analysis and describe how uncertainty about analytical judgements, inputs or projections affect the findings. Within this include any relevant proxy measures/methods to value the economic benefits.

**Discussion/conclusion**

- Explicitly describe who the ‘savings’ or economic benefits relate to.
- Report key findings, limitations, ethical or equity considerations not captured, and how these could affect patients, policy or practice.*
- Discuss the limitations associated with the proxy measures/methods to value the economic benefits.
- Discuss the generalisability or transferability of results across different settings and over time—particularly relating to the key parameters driving the ROI.

ROI has been commonly used in the private sector, especially in the business/investment field. That said, ROI is also increasingly being used to assess public sector healthcare investments.^3 4 6^ Within ROI the goal is to translate the benefits of an investment into a single quantitative measure expressed in monetary terms, so it can be directly compared with its cost. Within the business/investment sectors this is intuitive as the ultimate focus is usually focused on fiscal returns to an investor. However, it is less straightforward how ‘benefits’ should be defined and monetised in the context of public health interventions and there are a variety of non-fiscal benefits that could be potentially included.^6 7^

Although ROI analysis is closely related to cost-benefit analysis (as both of them aim to compare the cost and benefits of health interventions in monetary terms^4 8 9^), there is variation in the literature regarding whether studies estimating an ROI should be treated as a type or output of cost-benefit analysis, a separate type of full economic evaluation or a distinct type of health economic analysis looking at fiscal savings (online supplemental box 1).^2 4 9–15^ In addition, there is a particular form of ROI analysis known as social return on investment (SROI).^15–18^ Its framework goes beyond traditional health economic methods and considers the value produced for multiple stakeholders in three dimensions of development: economic, social and environmental.^16^

Although ROI analyses are increasingly being used to evaluate health interventions, this type of analysis is an area that has not been fully explored in the literature. A previous systematic review of the ROI of public health interventions was conducted by Masters *et al*.^4^ However, this study focused on examining the ROI values that have been estimated for existing public health interventions and the details of the specific methodology of the studies fell outside of the study’s scope. Given the potential role of the ROI metric for informing health policies, it is important that there is a comprehensive understanding of the uses of ROI analyses (such as the settings they are being used in and the health areas investigated), the terminology used to describe them and their methodology (such as the types of economic benefits being included and how they are being valued).

To address this need, we conducted a scoping review to investigate the range of uses, terminology and methodology within recent studies reporting the ROI metric to evaluate a health intervention. Due to the potentially large number of published ROI studies, we focused on gaining a comprehensive overview of the key features/methodology and reporting practices of recent studies (published between 2018 and 2021) rather than a review of the whole ROI literature. We focused on the following questions;

- What are the study settings and health areas where the ROI metric is being applied?
- What terminology is being used to describe the use of the ROI metric within such studies?
- What are the key methodological features of the ROI calculations and how well are they reported?
- What economic benefits are included within the ROI calculations (ie, does they only include fiscal savings) and what are the main methods used to monetise health benefits?

Investigating and reporting these features of ROI calculations will lead to greater awareness of how these analyses should be used to inform policy decisions and reduce the risk of the ROI metric being misinterpreted/misused. We also set out recommended use cases for ROI analysis and a reporting checklist for future studies.

## Methods

In line with the criteria outlined by Arksey and O’Malley we conducted a scoping review of studies reporting an ROI metric evaluating a health intervention within the public sector.^19^ Note that the goal was to identify the terminology and methodology surrounding studies reporting the ROI metric relating to a health intervention rather than determining if the actual type of analysis used matched what the paper claimed it to be.

### Search strategy and selection criteria

The publications were collected by searching the MEDLINE (via OVID), Embase Classic+Embase (via OVID), PubMed and Econ Lit databases on 14 June 2021. The search terms used in the database searches were (health OR healthcare) and ‘return on investment’ within the abstract and title field (see online supplemental information). The database search was limited to studies published between 1 January 2018 and 14 June 2021. This limited search period was chosen such that it would be feasible to comprehensively investigate the studies identified (capturing key methodological information) and that a reasonable sample would be found. No review protocol was published.

We included research articles that reported they were estimating the ROI of a health intervention within the public sector. This includes studies reporting an ROI metric even if it was not directly referred to as an ROI analysis (such as when a cost-benefit analysis estimates an ROI metric). The following criteria were used to exclude literature; non-English publications, reviews/systematic reviews, studies relating to corporate health workplace wellness programmes, studies relating to the education and training of healthcare professionals/students, conference abstracts and interventions on non-human animals.

The retrieved citations were uploaded to Covidence, a web-based systematic review software,^20^ to identify and remove duplicates. The screening was performed by a single reviewer—with consultation with a second reviewer to resolve any uncertainties.

### Data extraction and output

The relevant data (outlined in table 1) were extracted and input into a summary table in Excel.^21 22^ The data extraction was conducted by two reviewers independently. If more than one type of analysis was conducted within the same paper, we focused on the data/information, pertaining to the ROI calculations.

**Table 1:** Summary of the data extraction items

Setting and area investigated*	
Country setting	The country (or geographical area) where the analysed intervention was carried out was extracted. The income setting of the country/area studied was classified in accordance with the World Bank’s grouping.^21^
Health area(s) investigated	The health area(s) in question investigated were extracted and grouped into key categories (listed in online supplemental table S1). The chosen categories were adapted from those used by Pitt *et al* 22, with adding an additional category for smoking-related diseases. If more than one health area was investigated within the same study, then the study was counted in each relevant category. When the study was not related to any of the categories it was listed as other/ unclassifiable.
How the analysis was described and reported	
How the analysis was described	How the analysis was described was extracted, eg, cost-benefit analysis, ROI analysis, SROI analysis or evaluation of cost savings. If the description fitted across multiple categories, then it was counted in each relevant category. In addition, if the study was referred to as an ‘economic evaluation’ this was also extracted.
How are the ROI results were described	Information related to how the ROI results were described was extracted, ie, if the ROI was expressed as a ratio, as a percentage, only as a qualitative description (ie, high, positive or negative), or only as a numerical value. If the results were described in more than one way (ie, both a percentage and ratio), then the study was counted in each relevant category.
Key features	
Cost perspective	The cost perspective is the viewpoint applied to the economic analysis and affects the costs and outcomes that are included. If it was clearly reported, the perspective used was extracted. This was primarily based on searching the full text of the papers for the words ‘perspective’ and ‘viewpoint’. If more than one perspective was used within the same study, each one was extracted and counted separately.
Discounting	Discounting is a process used within health economic studies to convert costs and outcomes occurring in the future to their present value (reflecting the belief that, in general, society prefers to receive benefits sooner rather than later, and pay costs later rather than sooner). We recorded whether or not the studies reported that discounting was conducted.
Time horizon	The time horizon is the duration over which the costs and outcomes of the interventions are quantified. If the reported baseline time horizon(s) was clearly reported it was extracted. If a study reported multiple baseline time horizons, each one was extracted and reported separately.
Comparator	The comparator (or counterfactor) is an alternative scenario against which the intervention investigated is compared. Having a comparator is essential for the study to be a full economic evaluation. If the comparator used was clearly reported it was extracted. This was primarily based on searching the full text for the words ‘comparator’, ‘counterfactual’, ‘comparison’ and ‘compared to’.
What and how were the economic benefits calculated	
Types of economic benefits calculated	Health interventions and policies have a wide range of benefits that can be captured within ROI studies. The types of economic benefits that were included within the identified studies were extracted. We also extracted whether the study appeared to have investigated only fiscal/tangible savings (fiscal/ tangible costs were defined here as actual realisable financial monetary benefits to payers or society).
How were the health benefits monetised	A range of methods are used to translate health benefits into monetary terms to evaluate an intervention’s economic benefits. If health benefits were monetised, and the method used was extracted. If more than one method was used within the same study then the study was counted in each of the relevant categories.

*Due to the variation in terminology, information related to the type of interventions investigated was not extracted.

ROI, return on investment; SROI, social return on investment.

A Preferred Reporting Items for Systematic review and Meta-Analysis checklist is provided in the online supplemental information.^23^

## Results

**Figure 1:**
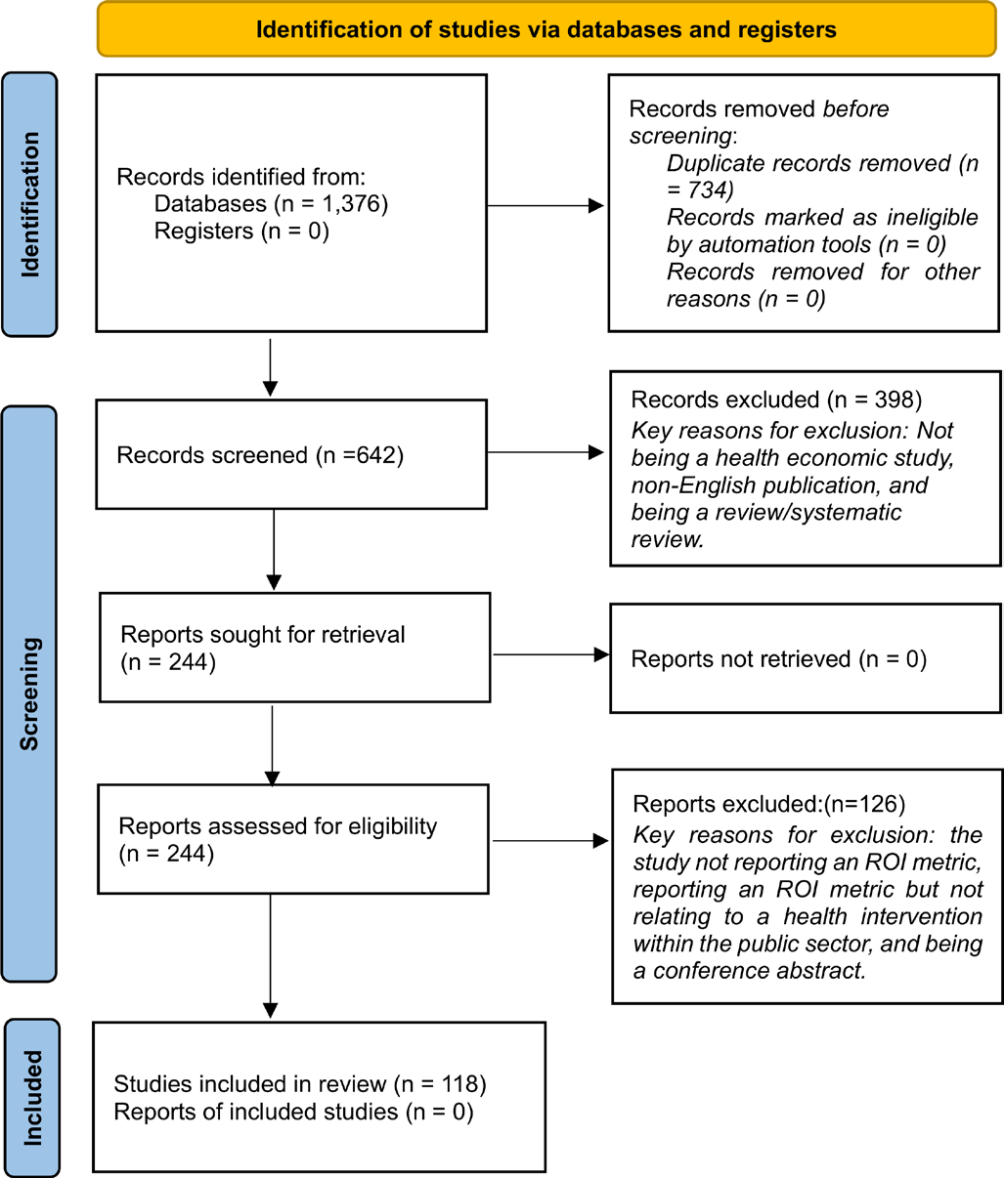
Flow diagram outlining the inclusion and exclusion of the identified studies. A Preferred Reporting Items for Systematic review and Meta-Analysis checklist is provided in the online supplemental information Supporting Information.^23^ ROI, return on investment.

We identified 1376 potentially relevant studies through database searches (figure 1). After removing duplicate papers in Covidence, a total of 642 studies remained. By conducting tile and abstract screening, 398 papers were excluded. The remaining 244 studies underwent a full-text screening, and after the further exclusion of 126 papers, 118 relevant studies were included within this scoping review. The summary of search results is described in figure 1.

Over our review period (2018–2021), there was no obvious trend in the number of ROI studies being published over time (online supplemental table S2).

### The setting and health area investigated

In terms of study setting, 55 (47%) of the studies were related to the USA (online supplemental table S3). In terms of the distribution across World Bank’s income groups,^21^ 80% of the studies related to high-income countries and 9% and 6%, related to upper-income and lower-middle-income countries, respectively (table 2).

**Table 2:** Overview of the settings/health areas investigated and descriptions of the analyses

Feature	Total	%
World Bank income setting		
High	94	80
Upper-middle	11	9
Lower-middle	7	6
Multiple countries—low-income and middle-income countries	4	3
Multiple countries—unclassifiable	2	2
Health areas investigated*		
Other/unclassifiable	22	19
Respiratory diseases	17	14
Cardiovascular diseases	13	11
Diabetes	12	10
Cancer and other neoplasms (excluding smoking-related diseases)	10	8
Smoking-related diseases	9	8
Mental health, cognition and developmental and behavioural disorders (including self-harm and substance disorders)	7	6
Malnutrition (including obesity and exercise)	6	5
HIV/AIDS	4	3
Neonatal and maternal conditions	3	3
Other infectious diseases (including encephalitis, hepatitis, other parasitic and vector-borne diseases and nematode infections)	3	3
The primary description of the analysis used in the study		
ROI/ROI analysis	69	58
Cost-benefit analysis	15	13
Social return on investment (SROI)	10	8
Cost-effectiveness analysis	10	8
Cost/programme savings	8	7
Economic/cost analysis	5	4
Investment case	3	3
Economic returns/benefit	2	2
Not clear	5	4
Mentions ‘economic evaluation’ within the text		
Yes	23	19
No	95	81
How the results of the ROI were presented		
As a ratio	83	70
As a percentage	29	25
Only the net amount of savings	4	3
Only with a qualitative description	3	3

For studies where multiple categories applied, the study was counted in each. Therefore, some studies are counted more than once under particular features, and the percentage breakdowns do not always add up to 100%.

*The categories were adapted from those used by Pitt *et al* 22 but adding an additional category for smoking-related diseases (online supplemental table S1). When the study was not related to any of these specific health areas (such as interventions to improve palliative care or community health worker programme) it was listed as other/unclassifiable.

ROI, return on investment.

The studies investigated a wide range of different health areas/topics (table 2). The most common specific areas investigated included respiratory diseases, cardiovascular diseases, diabetes, cancers and smoking-related diseases. A notable number also investigated non-specific forms of patient care not linked to a particular health area (such as palliative care or community health worker programmes). These are represented within the other/unclassified category of the investigated health area breakdown in table 2.

### How the ROI metric was described and reported

There was notable variation in how the studies reporting an ROI metric of an intervention described this analysis (table 2). The term ‘ROI analysis’ or terms like assessing an intervention’s ROI was used to describe 68 (58%) of the studies and 10 (8%) were described as SROI. The terms cost-benefit analysis (13%), or cost-effectiveness analysis (8%) were also used. Other terminology included an evaluation of cost/programme savings (7%), an economic/cost analysis (4%), an investment case (3%) and an evaluation of economic returns/benefit (2%). Only 23 (19%) studies explicitly mentioned the term ‘economic evaluation’ in their main text.

In terms of how the results were presented, a ratio was used for the majority of the studies (70%) (table 2). However, how the ratio was calculated was variable and in some cases, benefit-cost ratios appeared to be used instead (where the economic benefits rather than net benefits are divided by the cost of the intervention). The second most common description was the use of percentages, seen in 25% of the studies. Just 4% reported only a numerical value of the net savings/benefits without reporting any ratio or percentage.

### Reporting of key features of the analyses

A range of perspectives was used within the studies (table 3). However, in 48% of the studies, the perspective was not clearly reported. Five of the studies reported the results using multiple perspectives, such as both the payer and societal perspectives.

**Table 3:** Key features of the analyses and the types of benefits included

Feature	Total	%
Perspective		
Societal/social	23	19
Healthcare provider/system	19	16
Payer	12	10
Other	10	8
Unclear	60	48
Discounting included		
Yes	47	40
Not needed (time horizon under 1 year)	27	23
Unclear or not performed	44	37
Comparator type		
Without the intervention/no intervention/doing nothing	24	20
Control group	21	18
Status quo/usual care/current practice	19	16
Baseline/preintervention	18	15
Deadweight	8	7
Unclear/no comparator	28	24
Appears to only look at fiscal/tangible cost savings		
Yes	70	59
No	48	41
Valued health benefits		
Yes	48	41
No	70	59
How the health benefits were being valued*		
Productivity gains	19	40
Willingness to pay based metrics (including value per statistical life and the full-income approach)	16	33
Valuing DALY averted or QALYs gained†	6	13
Tax revenue	3	6
Other	2	4
Unclear	11	23
Included benefits other than monetised health gains and healthcare costs		
Yes	29	25
No	89	75

For studies where multiple categories applied, the study was counted in each. Therefore, some studies are counted more than once under particular features, and the percentage breakdowns do not always add up to 100%.

*Outlined further in online supplemental box 2.

†When relevant also counted under willingness to pay based metrics category.

DALY, disability-adjusted life year; QALY, quality-adjusted life year.

In terms of discounting, it was only clearly reported to be performed in 47 studies (40%). In 27 studies (23%) it would not be necessary as the time horizon was under 1 year and it was unclear whether or not it was performed in the remaining 44 studies (37%).

The baseline time horizon within the studies varied between 10 weeks to a lifetime. Time horizons between 1 and 5 years were used in 70 of the studies, and lifetime time horizon was used in 13 of the studies (online supplemental table S4). Overall, 17 (14%) of the studies did not clearly specify their time horizon.

A range of terminology was used to describe the comparators (table 3). A notable number of studies (24%) did not clearly mention the comparator within the text. The SROI studies tended to use a deadweight (the percentage of the return that would have occurred even without the intervention), instead of a formal comparator scenario.

Note that the proportion of items not clearly reported was notably higher for studies that were described as ROI/ROI analysis—particularly when compared with studies that calculated an ROI metric but were described as cost-benefit analysis or cost-effectiveness analysis (online supplemental table S5).

### The types of economic benefits that were included

The economic benefits included within the ROI calculations varied notably across the different studies (table 3). Seventy of the studies (59%) appeared to only quantify fiscal/tangible savings. Forty-eight of the studies (41%) valued health benefits in some way and 29 (25%) included economic benefits other than healthcare revenue, averted healthcare costs and monetised health gains These benefits were highly variable and specific to the context of the study. Examples of these other types of economic benefits included valuing education benefits, averted household costs, averted social care costs, tax revenue/government transfers, tourism revenue and monetised benefits related to improved well-being and averted grieving costs. The type of study influenced the types of economic benefits that were included and the SROI analysis tended to have an even broader range of benefits, and were more likely to include monetised social factors (such as improved self-esteem/well-being, reduced stress, improved attitude, increased support from family and increased confidence or knowledge). Table 3 lists the most common methods used within the studies to value health benefits monetarily where applicable (summarised in online supplemental box 2). Of the 48 studies that valued health benefits the valuation of productivity gains was used in 19 (40%) and a willingness to pay based metric was used in 16 (33%) of them. Six (13%) of these studies monetised the number of disability-adjusted life years (DALYs) averted or quality-adjusted life years (QALYs) gained. A small number of the studies adjusted for future income growth, and some adjusted for employment rates whereas most others did not adjust for either. For several of the studies, the methods were unclear and details of exactly how the benefits were valued were also not included/justified.

Although three of the studies considered the impact of the intervention and subsequent health gains on tax revenue, none fully quantified the macroeconomic impacts on gross domestic product (GDP)/GDP growth (such as with growth regressions or computable general equilibrium models measuring the macroeconomic impact of health indicators on GDP growth^24^). The closest to doing this were the two studies that used the full income approach (online supplemental box 2), which combines the value individuals place on increased life expectancy with changes in a measure of national income growth (such as the GDP),^25^ but this only captures the immediate effect of mortality on labour supply and not the wider knock-on effects.

## Discussion

This scoping review is the first to comprehensively investigate the uses of ROI analysis, how they are described and their methodology. It demonstrates that there is notable variation in the methodology surrounding recent ROI analyses of health interventions, as well as the actual definition of an ROI analysis being used. This methodological variation is important as it means that studies reporting an ROI are often not directly comparable to one another. Such variation also risks generating systematic biases in how studies are conducted and interpreted, with approaches that generate higher ROI potentiality being favoured by some studies. This makes it difficult for decision-makers to plan investments based on interventions with the highest ROI, given that they may be based on studies with non-comparable methodology and could result in suboptimal decisions.

We also found that many of the studies identified within this scoping review did not explicitly clarify important methodological components, and there were notable inconsistencies regarding how the analyses were defined and the methods used. For example, some of the studies reporting an ROI appeared to be calculated benefit-cost ratios (where the economic benefits are divided by the cost of the intervention) rather than the traditional ROI calculations. These findings have implications regarding distinguishing between different types of health economic studies. The variation in the use of ROI metric (such as some studies only including fiscal savings vs others including monetised health and non-health benefits) highlights the difficulty in having a formal universal definition distinguishing between ROI and cost-benefit analysis (both of which express outcomes in monetary units). Some could have conceivably distinguished ROI analyses from other types of health economic studies by including and monetising non-health benefits. However, only 25% of the identified ROI studies monetised non-health benefits, and these benefits can be included in cost-benefit analyses^26 27^ and even in a cost-effectiveness analysis when using the societal perspective.^28^ Thus, the inclusion of monetised non-health benefits does not formally distinguish ROI from cost-benefit analysis. An alternative definition could be that ROI only quantifies the fiscal/financial returns from an intervention. However, our findings show that non-fiscal costs are sometimes included, and therefore this definition also does not universally apply. Consequently, the definition being used, and the types of cost included will likely depend on the specific study and it is important not to overgeneralise terminology.

Over our review period (2018–2021), we did not observe an increasing trend in the number of ROI studies being published over time. That said, the number of studies published between 2020 and 2021 was likely influenced by the COVID-19 pandemic.

In terms of uses, we found that the ROI metric is being applied in a wide range of study settings and health areas. That said, 47% of the studies were conducted for the USA and 80% of the studies were related to high-income countries. Compared with the findings of a bibliometric analysis of the economic evaluations,^22^ the proportion of studies across the different World Bank income settings were similar, but we found a higher proportion of studies relating to the USA (47% vs 35%). This trend could be partly due to the fact that in the USA, the Affordable Care Act prohibits the Patient-Centered Outcomes Research Institute from using cost-per-QALY benchmark to establish what type of healthcare is cost effective or recommended^29^ (potentially increasing the reliance on other metrics, such as ROI). A further factor that could be influencing this distribution across study settings is that investment cases for interventions in low and middle-income countries (LMICs) may be published more in the grey literature (such as^30–32^) and therefore not detected by our literature search. That said, as investment cases become more common, this trend will likely change, and more ROI studies will be conducted in LMICs. The broader reasons and implications of health economic studies being less represented in LMICs are outlined in Pitt *et al*.^22^ Regardless of the setting, this study highlights the need for the development of methodological guidelines and reference cases to ensure the quality and comparability of future ROI studies.

There was notable variation in how the studies reporting an ROI metric of a health intervention described the analysis. Crucially many studies did not clearly report the comparator, which has implications regarding whether the study is a full economic evaluation or not (online supplemental box 1). This is notable as although ROI analysis is increasingly being referred to as a type of full economic evaluation, this review indicates that this will not always be the case.^12 13^ Importantly, studies that ignore the comparator/relevant policy alternatives can generate misleading conclusions (online supplemental box 1). The terminology related to the comparators being used (table 3) was more variable than the comparators typically recommended within economic evaluation guidelines.^33^

A related factor was the variation regarding what the ‘purpose’ of the ROI analysis was (including if they were considering technical efficiency vs allocative efficiency) and the corresponding targeted audience they were seeking to inform. For example, within the studies we identified many were using the ROI metric to evaluate if a particular intervention/policy would generate fiscal cost savings (ie, an accounting tool/exercise), some were using it in an investment case context to justify continued or greater resources/funds for an intervention, and some using it as an output of a formal economic evaluation of the costs and benefits of alternative interventions/strategies aimed at informing the optimum policy option. However, this purpose was not always clear. This indicates that there is variation regarding what type of analysis ROI studies actually are when applied within the healthcare sector—which needs to be understood when interpreting these studies. Importantly, not all ROI analyses will be a formal economic evaluation.

### Overview of key findings: the reporting of key methodological features of the ROI calculations

A notable number of the identified studies do not clearly report key features of the methodology of ROI calculations in sufficient detail. For example, 48% of studies did not explicitly report the perspective of the analysis, the comparator was not clearly stated in 24% of studies, and the time horizon in 14%. These features of economic analysis need to be explicitly reported within studies, and without this, it makes it very difficult to formally compare the results of the different studies.^1 34^ In addition, the methods used to value the economic benefits within the ROI calculations were also not always clearly reported. For example, for 11 (23%) of these 48 studies that included the monetised value of health benefits, the methods were unclear and details of exactly how the benefits were valued were also not included/justified.

Although poor reporting has been observed for economic evaluations more generally,^35–37^ it appeared more extreme within this sample of ROI studies. For example in a review of cost-per-DALY averted studies between 2000 and 2015,^36^ a ‘Not stated/other’ perspective category was only found in 2% of the studies, and the discount rate for the costs could not be determined in 17% of the studies. This could partly be explained by the different purposes of analysis and the fact that not all of these ROI studies were actual full economic evaluations as many lacked formal comparators (online supplemental box 1). Interestingly, the studies that were referred to as types of economic evaluations (such as cost-benefit analysis or cost-effectiveness analysis) tended to have better reporting quality (online supplemental table S5). The Consolidated Health Economic Evaluation Reporting Standards (CHEERS) statement summarises recommendations to improve the reporting style of health economic evaluations.^38^ The latest update has had the language broadened to make the checklist more widely applicable (such as for cost-benefit analysis).^38^ However, although a useful tool, it is still specific to economic evaluations and is therefore not typically applied to non-economic evaluation studies. In addition, the CHEERS checklist is a reporting guidance and does not assess methodology quality. Therefore, there are still specific features of ROI calculations that need better assessment which is outside the scope of the CHEERS checklist. It is important that further methodological guidelines and reference cases for ROI studies are developed.

### Overview of key findings: the economic benefits included

One of the key and most significant inconsistencies across these ROI studies was regarding what type of economic benefits were quantified. The economic benefits included varied, including averted healthcare costs, monetised health benefits, as well as education benefits, changes in tax revenue, tourism revenue and averted intangible costs related to grieving. Crucially, ROI analysis in public health can extend beyond quantifying benefits in terms of financial returns and cost savings to monetise other factors/sources to consider their ‘value’—such as health benefits. Consequently, many ROI estimates not only look at the medical cost savings that result from a health intervention but are evaluating a broader range of monetised benefits. Part of this variation in the benefits included will be due to the different perspectives used across the analysis (healthcare provider, government vs the broader societal perspective) as well as its corresponding purpose/foundation of the analysis, that is, in some cases it is an accounting based tool for analysing/modelling fiscal cost savings and in some cases an output of an economic evaluation. This underlines the need to clearly specify the study’s perspective, however 48% of the studies did not do this.

The variation regarding if the study is quantifying only fiscal/tangible cost savings or broader non-fiscal benefits is important as it changes how the ROI metric should be interpreted. Many could interpret an intervention having an ROI of 1000%, as generating US$10 in fiscal benefits to the health system or society for every US$1 spent on it—like in a business/investment context. However, this is not the correct interpretation when non-fiscal/intangible economic benefits are included.^8^

In addition, as well as variation in what benefits were included there were also differences in the methods used to convert health benefits into a monetary value. The main methods used included valuing productivity gains, using willingness to pay measures (such as the value of a statistical life) and converting QALY/DALY health measures to monetary values using a threshold. These different approaches have different theoretical foundations (eg, welfarist vs extra-welfarist) and can lead to variations in the outcome of the economic study and correct interpretation.^9 39^ For example, measuring benefits based on the value of a statistical life often generates larger economic benefits than measuring benefits based on productivity gains.^39^ This is because the former includes both financial benefits (such as medical expenses and losses of future income) and non-financial benefits (such as avoided pain) of the health intervention, while the latter only focuses on lost production.^40 41^ A summary of the limitations of both the valuing of productivity gains and willingness to pay approaches for monetising health benefits is provided by Turner *et al*.^2^ Many of the studies did not provide details of the methods used. This is concerning as even if just valuing productivity gains, it is possible to get notably different answers depending on the specific approach being used. For example, some of the studies adjusted for future wage growth whereas others did not—potentially leading to significant differences.

The SROI analyses reviewed included a broader range of benefits (monetising social factors such as improved self-esteem, increased support from family, improved attitude and increased confidence or knowledge)—which are monetised to give a single quantitative measure of the benefits. However, many of these broad benefits included within these SROI studies can be difficult to monetise, and therefore the results will be highly dependent on what approach has been used and will likely vary across different studies. It is important this is understood when interpreting and comparing these studies.

### Limitations of this study

Although the methodology used for this scoping review was appropriate to achieve the aims of this study, there are limitations that are important to acknowledge.

The included publications were limited to papers written in English, and searches were limited to publications related to health interventions within the public sector published between 1 January 2018 and 14 June 2021. This limited search period was chosen to allow us to comprehensively investigate the identified studies. It was not possible to use a longer time frame due to the number of papers that would have been found as well as the time needed to extract the information needed. We believe that, overall, our findings are robust to this limitation as we included 118 recent studies. In addition, although several databases were searched, some relevant studies may not have been detected and we did not include grey literature. However, the overarching findings of this scoping review would be robust to these limitations.

Furthermore, it is important to note that only one researcher performed the screening. However, there were discussions with a second reviewer to resolve any conflicts/uncertainties, and two reviewers did the data extraction.

For this review, our goal was to identify inconsistencies in the terminology and methodology surrounding studies reporting the ROI metric. We therefore chose to provide descriptive information from a broader range of studies rather than performing a detailed evaluation of each study identified. Due to this, the methodological quality of each included study was not assessed, and we did not extract specific results/conclusions from the identified ROI analysis. In addition, details surrounding how the benefits other than monetised health gains and averted healthcare costs were valued were not evaluated in detail. These benefits were highly variable and contextual. The methodology surrounding the inclusion and valuation of these benefits is an area that needs further attention and research.

### Implications and policy recommendations related to the use of the ROI metric

We found that ROI analyses are used for multiple purposes within the healthcare sector (including evaluation of cost savings, advocacy/investment cases and as an output of economic evaluations). Although there are important advantages of the ROI metric (such as being intuitive to interpret from a range of stakeholders), it may not always be an appropriate analysis/metric for all of the purposes it is being used for.

We found that currently, ROI calculations of health interventions are using inconsistent methods, and they are often poorly reported. This is not an ideal combination in the context of using these studies to inform policy decisions, particularly when evaluating the value for money of different interventions, as there is always going to be a need to compare different health economic analyses informing resource allocation. Moving forward, to improve the inconsistencies in ROI analysis as well as to ensure proper reporting, it is advisable that guidelines on how to report and conduct ROI analysis are developed. We would recommend that these guidelines should specify that studies explicitly state how the benefits are being valued monetarily, clearly stating if the costs relate to fiscal/tangible benefits or not. This is important given their corresponding use in the business/investment sector and for analysis of fiscal cost-savings, there is a risk that stakeholders could misinterpret the results and assume that studies are reporting fiscal/tangible cost-savings (either to their health system or their society as a whole), when in fact in some studies a large proportion of the estimated economic benefits will not be directly realisable/are non-fiscal.^8^ This potential for misinterpretation is an important limitation of ROI analysis. In box 1 we outline proposed recommendations for the reporting of future ROI studies.

Public health aims to improve the health of the population rather than saving money. In the context of informing the optimum allocation of healthcare resources, it is also important to consider that although health interventions having a positive ROI in terms of fiscal savings is obviously advantageous it should not necessarily be the primary focus (particularly when considering the allocative efficiency of resources within the health sector). An intervention may not generate a positive ROI when only evaluating fiscal/tangible benefits but that does not mean it would not be a cost-effective intervention in terms of the health gains it generates, that is, just because it does not generate cost-savings it does not mean the national health system should not adopt the intervention. This is not to say that there are no important advantages of ROI analysis and we are not denying it can be useful or there are no well-conducted ROI studies.^2^ However, based on our findings and the variation between the different studies, we advise a degree of caution in using the ROI metric for policy decisions. Our recommended use cases for ROI analysis are outlined in box 1. Specifically, we would argue that the ROI metric is useful in the context of investigating fiscal cost savings (ie, as an accounting tool) of new interventions and for advocacy purposes (such as an investment case helping to raise/safeguard funding for public health programmes) (box 1).^6^ In contrast, in the context of health technology assessment/priority setting for health benefit package development,^42 43^ we would argue that traditional economic evaluations will typically be more appropriate than ROI approaches, due to the risk of the misinterpretation of the ROI-based outputs and the notable variation in the methodology employed in their calculation. This could change with the development of national/international ROI to improve the consistency of methodology.

In addition, the ROI metric is often used in the context of ‘investment cases’—which aim to articulate the need for specific investments in health (ie, an advocacy tool). Lauer *et al* 9 also highlighted the risk of dressing up a cost-effectiveness analysis as an investment case (such as monetising the health benefits) and argued that an investment case should report at least some market-valued benefits in order to so qualify as such. A scoping review of investment cases for vaccines and immunisation programmes by Sim *et al* 44 also concluded that the field was inconsistent and needed guidelines.

## Conclusion

We found that there was a fundamental variation regarding whether published ROI calculations of health interventions included only fiscal savings (such as prevented medical expenses) or a wider range of benefits (such as monetised health benefits). This highlights the variation in the definition of ROI analyses and supports the finding that ROI analyses are used for a range of different research questions/purposes within the healthcare sector. It is therefore important that how it is being used in one particular health area/setting or one particular definition is not overgeneralised. We also found these ROI calculations used inconsistent methodologies and were often poorly reported. This is a particularly important limitation in the context of priority setting, as it is vital to be able to compare studies assessing different interventions accurately. This is not to say that there are no important advantages of ROI analysis, and we are not denying it can be useful or that there are no well-conducted ROI studies. However, based on the variation in its current usage and methodology we recommend a degree of caution using the ROI metric in the context of health technology assessment/priority setting for informing policy decisions surrounding the allocation of healthcare resources.

## Data Availability

Data are available upon reasonable request. Data are available upon reasonable request to the corresponding author.
