# Supplementary material for: Analyses of the return on investment of public health interventions: a scoping review and recommendations for future studies
Source: BMJ Glob Health. 2023 Aug 30;8(8):e012798. doi: 10.1136/bmjgh-2023-012798 (PMC10471881; doi:10.1136/bmjgh-2023-012798)
Supplement: Supplementary data [file bmjgh-2023-012798supp001.pdf]

*Analyses of the return on investment of public health interventions: A scoping review and recommendations for future studies*

# Supporting information

## Analyses of the return on investment of public health interventions: A scoping review and recommendations for future studies

Hugo C Turner[ł1*], Yoshiaki Hori[ł2], Paul Revill[3], Waranya Rattanavipapong[4], Ko Arai[5], Justice Nonvignon[6], Mark Jit[8,9], Yot Teerawattananon[4,10]

[1] MRC Centre for Global Infectious Disease Analysis, School of Public Health, Imperial College London, London, UK.
[2] School of Public Health, Imperial College London, London, UK.
[3] Center for Health Economics, University of York, York, UK.
[4] Health Intervention and Technology Assessment Program (HITAP), Ministry of Public Health, Nonthaburi, Thailand.
[5] Graduate School of Business Administration, Hitotsubashi University, Tokyo, Japan
[6] Africa Centres for Disease Control and Prevention, Addis Ababa, Ethiopia
[7] School of Public Health, University of Ghana, Legon, Ghana
[8] Department of Infectious Disease Epidemiology, London School of Hygiene and Tropical Medicine, London, UK.
[9] School of Public Health, University of Hong Kong, Hong Kong Special Administrative Region, China.
[10] Saw Swee Hock School of Public Health, National University of Singapore, Singapore.

ł Joint first authors

* Corresponding author

*Analyses of the return on investment of public health interventions: A scoping review and recommendations for future studies*

**Pubmed search terms**

("return* on investment*"[Title/Abstract] AND ("health"[Title/Abstract] OR "healthcare"[Title/Abstract])) AND (2018/1/1:2021/6/14[pdat])

An asterisk (*), used at the end of a word, was to specify any number of characters for variable endings of a root word

*Analyses of the return on investment of public health interventions: A scoping review and recommendations for future studies*

**Supporting Box 1: Health economics analysis: partial evaluations versus full economic evaluations**

The purpose of health economics is to inform optimal resource allocation to improve the population's health. Health economic analysis and evaluation can be defined as studies that fall under this remit and they are an integral part of the development of health policy. In the context of evaluating health interventions, there is a distinct type of health economic analysis known as full economic evaluations.

A full economic evaluation is a specific type of health economic analysis that explicitly compare both the costs (use of resources) and consequences (effects) of the interventions in question with an alternative course of action, known as the comparator [1]. Full economic evaluation includes cost-effectiveness analysis, cost-utility analysis, cost-benefit analysis, cost-minimization analysis, and cost-consequence analysis.

In contrast, a partial evaluation is a type of health economic analysis that only examines either the costs or consequences of an intervention independently (i.e. they do not look at both or relate the costs to the consequences), or a study that evaluates both the costs and consequences of an intervention but only evaluates a single course of action (a cost outcome study) [2,3].

Importantly, without an appropriate comparator, an evaluation of the benefits and costs of a new intervention can be misleading. For example, the economic benefits of a new intervention may outweigh its costs. However, it is important to compare this to the current practice in analysis. It is possible for a new intervention to have a positive ROI when ignoring potential alternative options, however, it could still be less effective and more costly in comparison to the current practice. Therefore full economic evaluations are more useful for decision making surrounding resource allocation, as they consider both the costs and benefits of interventions, and account for alternative policy options [3].

*Analyses of the return on investment of public health interventions: A scoping review and recommendations for future studies*

**Supporting Box 2: Examples of ways health benefits were monetised in ROI studies**

Valuing productivity gains: Productivity gains were valued in some ROI studies by placing a monetary value on the estimated productivity losses associated with a disease that are averted due to a health intervention. This often involved valuing a patient's time based on their wages.

Willingness to pay metrics: The willingness to pay technique is based on the premise that the maximum amount an individual is willing to pay (or sacrifice) for a given commodity is an indicator of its 'value' to them [4]. Using the willingness to pay technique in this context, some studies estimated what an individual is willing to pay for certain health benefits, consequently estimating the value of the health benefits of an intervention in monetary terms for that individual. An advantage of this is that it is argued that when an individual is considering their maximum willingness to pay, they will take account of all the attributes of the service of importance to them, not just the health gains [4]. An example of a willingness to pay metric is the "value per statistical life" (VSL) which captures how much individuals are willing to pay to reduce the risk of death and is used to estimate a monetary value on reductions in mortality (i.e. the number of deaths averted by a health intervention). This is often based on trade-offs individuals are willing to make between fatality risk and consumption. This can also be expressed as a "value per statistical life year" (VSLY). The full income approach taken by some studies combined the value people place on increased life expectancy (i.e. valuing additional life-years using VSLY metrics) to changes in a measure of national income growth (such as the gross domestic product (GDP)) [5].

Converting health benefits metrics with a thresholds: Another method was to use a threshold to convert health metrics (such as DALY averted or QALYs gained) into a monetary value [6]. This approach can use willingness to pay metrics for the conversion.

*Analyses of the return on investment of public health interventions: A scoping review and recommendations for future studies*

**Supporting Table S1: Categories of the different health areas**

| |
|---|
| Anaemia |
| Cancer and other neoplasms (excluding smoking related diseases) |
| Cardiovascular diseases |
| Communicable childhood diseases |
| Congenital anomalies |
| Diabetes |
| Diarrhoeal diseases |
| Digestive disorders |
| Endocrine, blood, and immune disorders (excluding diabetes or HIV) |
| Genitourinary diseases, contraception & fertility |
| HIV/AIDS |
| Malnutrition (including obesity and exercise) |
| Meningitis |
| Mental health, cognition, and developmental and behavioural disorders (including self-harm and substance disorders) |
| Musculoskeletal diseases (including back pain) |
| Neonatal and maternal conditions |
| Neurological conditions |
| Other infectious diseases (including encephalitis, hepatitis, other parasitic and vector-borne diseases, and nematode infections) |
| Respiratory diseases |
| Sense organ diseases |
| Sexually transmitted diseases (excluding HIV) |
| Skin and oral conditions |
| Smoking related disease* |
| Tuberculosis |
| Unclassifiable |
| Wounds and injuries (including violence) |

*The chosen categories were adapted from those used by Pit et al. [7], with adding an additional category for smoking-related diseases.*

*Analyses of the return on investment of public health interventions: A scoping review and recommendations for future studies*

**Supporting Table S2: Publication trend of ROI studies**

| Year | Average number of ROI studies published per month |
|---|---|
| 2018 | 2.83 |
| 2019 | 2.50 |
| 2020 | 3.33 |
| 2021 | 2.15 |

*Analyses of the return on investment of public health interventions: A scoping review and recommendations for future studies*

**Supporting Table S3: The country settings**

| Country setting | Number of studies | % |
|---|---|---|
| USA | 55 | 47% |
| UK | 7 | 6% |
| Canada | 7 | 6% |
| Spain | 6 | 5% |
| Multiple countries | 6 | 5% |
| Thailand | 5 | 4% |
| Australia | 4 | 3% |
| China | 3 | 3% |
| Kenya | 2 | 2% |
| Germany | 2 | 2% |
| Italy | 2 | 2% |
| New Zealand | 2 | 2% |
| Jamaica | 1 | 1% |
| Timor Leste | 1 | 1% |
| Switzerland | 1 | 1% |
| Mexico | 1 | 1% |
| unclear | 1 | 1% |
| Hungary | 1 | 1% |
| Ireland | 1 | 1% |
| Vietnam | 1 | 1% |
| Greece | 1 | 1% |
| India | 1 | 1% |
| Denmark | 1 | 1% |
| Finland | 1 | 1% |
| Egypt | 1 | 1% |
| South Africa | 1 | 1% |
| South Korea | 1 | 1% |
| Nigeria | 1 | 1% |
| Saudi Arabia | 1 | 1% |

*Analyses of the return on investment of public health interventions: A scoping review and recommendations for future studies*

**Supporting Table S4: Baseline time horizon used in the analyses investigated**

| Time horizon (years) | Number of studies |
|---|---|
| 1 year or less | 34 |
| >1 to 2 years | 12 |
| >2-5 years | 24 |
| >5-10 years | 12 |
| >10-30 years | 11 |
| >30-70 years | 4 |
| Lifetime | 13 |
| Unclear | 17 |

*Studies that reported multiple baseline time horizons have each time horizon reported separately. Some studies had time horizons in their sensitivity analysis not reported here.*

*Analyses of the return on investment of public health interventions: A scoping review and recommendations for future studies*

## Supporting Table S5: Reporting of key features stratified by the description of the analysis used in the study

| Reporting of a key feature | The description of the analysis used in the study | | | | | | | | | |
|---|---|---|---|---|---|---|---|---|---|---|
| | **ROI/ROI analysis** | **CBA** | **SROI** | **CEA** | **Cost/programme savings** | **Economic/cost analysis** | **Investment case** | **Economic returns/benefit** | **Not clear** | **Total** |
| **Comparator** | | | | | | | | | | |
| Unclear | 21 | 1 | 1 | 2 | 0 | 1 | 0 | 0 | 2 | 28 |
| Clear | 47 | 14 | 9 | 7 | 8 | 4 | 3 | 2 | 4 | 98 |
| Total | 68 | 15 | 10 | 9 | 8 | 5 | 3 | 2 | 6 | 126 |
| **Perspective** | | | | | | | | | | |
| Unclear | 43 | 2 | 0 | 2 | 7 | 3 | 1 | 2 | 5 | 65 |
| Clear | 25 | 13 | 10 | 7 | 1 | 2 | 2 | 0 | 1 | 61 |
| Total | 68 | 15 | 10 | 9 | 8 | 5 | 3 | 2 | 6 | 126 |
| **Discounting** | | | | | | | | | | |
| Unclear | 33 | 1 | 0 | 1 | 6 | 4 | 0 | 0 | 4 | 49 |
| Clear/not needed | 35 | 14 | 10 | 8 | 2 | 1 | 3 | 2 | 2 | 77 |
| Total | 68 | 15 | 10 | 9 | 8 | 5 | 3 | 2 | 6 | 126 |
| **Time horizon** | | | | | | | | | | |
| Unclear | 13 | 0 | 0 | 0 | 0 | 1 | 0 | 1 | 2 | 17 |
| Clear | 55 | 15 | 10 | 9 | 8 | 4 | 3 | 1 | 4 | 109 |
| Total | 68 | 15 | 10 | 9 | 8 | 5 | 3 | 2 | 6 | 126 |

*Some studies had descriptions for analysis that fitted across more than one of the chosen categories (such as both a ROI and a CEA). In such cases, the study was counted in each of the relevant categories. Therefore, some studies are counted more than once.*
*CBA; Cost-benefit analysis, CEA; Cost-effectiveness analysis, SROI; Social return on investment, ROI; Return on investment*



Turner HC, *et al. BMJ Glob Health* 2023; 8:e012798. doi: 10.1136/bmjgh-2023-012798

*Analyses of the return on investment of public health interventions: A scoping review and recommendations for future studies*

**Supporting Table S6: Checklist for the reporting ROI studies in the health sector**

| Item | Area | Reported in section |
|---|---|---|
| | Introduction | |
| 1 | Give the context for the study, the study question, and its practical relevance for decision making in policy or practice.* | |
| 2 | Justify the purpose of the analysis, target audience and why ROI is an appropriate metric. | |
| | Methods | |
| 3 | Describe the characteristics of the study population.* | |
| 4 | Describe the interventions or strategies/scenarios being compared and why they were chosen (the comparator or counterfactual).* | |
| 5 | State the perspective(s) adopted by the study and outline why chosen.* | |
| 6 | State the time horizon for the study and outline why it is appropriate.* | |
| 7 | Report the discount rate(s) and outline why chosen.* | |
| 8 | Describe the specific ROI calculation being used (i.e., how is the ratio or percentage being calculated). | |
| 9 | Report all analytic inputs and parameters (such as values, ranges, references). Include a Table that lists which economic benefits are being included and explicitly how they are being valued monetarily. Clearly stating if the costs relate to fiscal/tangible benefits or not. | |
| | Results | |
| 10 | Provide a clear breakdown of the ROI stratified by the different types of benefits and stakeholders. | |
| 11 | Report the absolute numbers regarding the cost and benefits and not just the summary ratio/percentage.* | |
| 12 | Report the results stratified by including only fiscal/tangible benefits and non-fiscal. | |
| 13 | If including non-fiscal savings – avoid phrasing such as for every dollar invested generates the "US$X" value in returns. | |
| 14 | Perform a sensitivity analysis and describe how uncertainty about analytic judgments, inputs, or projections affect the findings. Within this include any relevant proxy measures/methods to value the economic benefits. | |
| | Discussion/conclusion | |
| 15 | Explicitly describe who the "savings" or economic benefits relate to. | |
| 16 | Report key findings, limitations, ethical or equity considerations not captured, and how these could affect patients, policy, or practice.* | |
| 17 | Discuss the limitations associated with the proxy measures/methods to value the economic benefits. | |
| 18 | Discuss the generalisability or transferability of results across different settings and over time – particularly relating to the key parameters driving the ROI. | |

*The following is a checklist for ROI studies in the health sector. We also recommend that economic evaluations should follow the Consolidated Health Economic Evaluation Reporting Standards (CHEERS) recommendations [9]. Note that some of these items were adapted from the CHEERS recommendations (indicated with *).*

*Analyses of the return on investment of public health interventions: A scoping review and recommendations for future studies*

## Preferred Reporting Items for Systematic reviews and Meta-Analyses extension for Scoping Reviews (PRISMA-ScR) Checklist

| SECTION | ITEM | PRISMA-ScR CHECKLIST ITEM | REPORTED ON PAGE # |
|---|---|---|---|
| **TITLE** | | | |
| Title | 1 | Identify the report as a scoping review. | 1 |
| **ABSTRACT** | | | |
| Structured summary | 2 | Provide a structured summary that includes (as applicable): background, objectives, eligibility criteria, sources of evidence, charting methods, results, and conclusions that relate to the review questions and objectives. | 2 |
| **INTRODUCTION** | | | |
| Rationale | 3 | Describe the rationale for the review in the context of what is already known. Explain why the review questions/objectives lend themselves to a scoping review approach. | 3-4 |
| Objectives | 4 | Provide an explicit statement of the questions and objectives being addressed with reference to their key elements (e.g., population or participants, concepts, and context) or other relevant key elements used to conceptualize the review questions and/or objectives. | 4 |
| **METHODS** | | | |
| Protocol and registration | 5 | Indicate whether a review protocol exists; state if and where it can be accessed (e.g., a Web address); and if available, provide registration information, including the registration number. | 5 |
| Eligibility criteria | 6 | Specify characteristics of the sources of evidence used as eligibility criteria (e.g., years considered, language, and publication status), and provide a rationale. | 5 |
| Information sources* | 7 | Describe all information sources in the search (e.g., databases with dates of coverage and contact with authors to identify additional sources), as well as the date the most recent search was executed. | 5 |
| Search | 8 | Present the full electronic search strategy for at least 1 database, including any limits used, such that it could be repeated. | Supporting information |
| Selection of sources of evidence† | 9 | State the process for selecting sources of evidence (i.e., screening and eligibility) included in the scoping review. | 5 |
| Data charting process‡ | 10 | Describe the methods of charting data from the included sources of evidence (e.g., calibrated forms or forms that have been tested by the team before their use, and whether data charting was done independently or in duplicate) and any processes for obtaining and confirming data from investigators. | 5-6 |
| Data items | 11 | List and define all variables for which data were sought and any assumptions and simplifications made. | Table 1 |
| Critical appraisal of | 12 | If done, provide a rationale for conducting a critical appraisal of | NA |

*Analyses of the return on investment of public health interventions: A scoping review and recommendations for future studies*

| SECTION | ITEM | PRISMA-ScR CHECKLIST ITEM | REPORTED ON PAGE # |
|---|---|---|---|
| individual sources of evidence§ | | included sources of evidence; describe the methods used and how this information was used in any data synthesis (if appropriate). | |
| Synthesis of results | 13 | Describe the methods of handling and summarizing the data that were charted. | 5-6 |
| **RESULTS** | | | |
| Selection of sources of evidence | 14 | Give numbers of sources of evidence screened, assessed for eligibility, and included in the review, with reasons for exclusions at each stage, ideally using a flow diagram. | Figure 1 |
| Characteristics of sources of evidence | 15 | For each source of evidence, present characteristics for which data were charted and provide the citations. | 5-8 |
| Critical appraisal within sources of evidence | 16 | If done, present data on critical appraisal of included sources of evidence (see item 12). | NA |
| Results of individual sources of evidence | 17 | For each included source of evidence, present the relevant data that were charted that relate to the review questions and objectives. | 5-8 |
| Synthesis of results | 18 | Summarize and/or present the charting results as they relate to the review questions and objectives. | 5-8 |
| **DISCUSSION** | | | |
| Summary of evidence | 19 | Summarize the main results (including an overview of concepts, themes, and types of evidence available), link to the review questions and objectives, and consider the relevance to key groups. | 8-14 |
| Limitations | 20 | Discuss the limitations of the scoping review process. | 12 |
| Conclusions | 21 | Provide a general interpretation of the results with respect to the review questions and objectives, as well as potential implications and/or next steps. | 14 |
| **FUNDING** | | | |
| Funding | 22 | Describe sources of funding for the included sources of evidence, as well as sources of funding for the scoping review. Describe the role of the funders of the scoping review. | 14 |

JBI = Joanna Briggs Institute; PRISMA-ScR = Preferred Reporting Items for Systematic reviews and Meta-Analyses extension for Scoping Reviews.

* Where *sources of evidence* (see second footnote) are compiled from, such as bibliographic databases, social media platforms, and Web sites.

† A more inclusive/heterogeneous term used to account for the different types of evidence or data sources (e.g., quantitative and/or qualitative research, expert opinion, and policy documents) that may be eligible in a scoping review as opposed to only studies. This is not to be confused with *information sources* (see first footnote).

‡ The frameworks by Arksey and O'Malley (6) and Levac and colleagues (7) and the JBI guidance (4, 5) refer to the process of data extraction in a scoping review as data charting.

§ The process of systematically examining research evidence to assess its validity, results, and relevance before using it to inform a decision. This term is used for items 12 and 19 instead of "risk of bias" (which is more applicable to systematic reviews of interventions) to include and acknowledge the various sources of evidence that may be used in a scoping review (e.g., quantitative and/or qualitative research, expert opinion, and policy document).

*From:* Tricco AC, Lillie E, Zarin W, O'Brien KK, Colquhoun H, Levac D, et al. PRISMA Extension for Scoping Reviews (PRISMAScR): Checklist and Explanation. Ann Intern Med. 2018;169:467–473. doi: 10.7326/M18-0850

*Analyses of the return on investment of public health interventions: A scoping review and recommendations for future studies*

# References

1. Drummond MF, Sculpher MJ, Claxton K, Stoddart GL, Torrance GW. Methods for the Economic Evaluation of Health Care Programmes. Oxford: Oxford University Press; 2015.
2. Morris S, Devlin N, Parkin D. Economic analysis in health care. 2nd ed. ed. Chichester: Wiley; 2012.
3. Turner HC, Archer RA, Downey LE, et al. An introduction to the main types of economic evaluations used for informing priority setting and resource allocation in healthcare: key features, uses and limitations. *Frontiers in public health "In Press"* 2021.
4. McIntosh E, Luengo-Fernandez R. Economic evaluation. Part 1: Introduction to the concepts of economic evaluation in health care. *The journal of family planning and reproductive health care* 2006; **32**(2): 107-12.
5. Jamison DT, Summers LH, Alleyne G, et al. Global health 2035: a world converging within a generation. *Lancet* 2013; **382**(9908): 1898-955.
6. Lisa A. Robinson JKH, Michele Cecchini, Kalipso Chalkidou, Karl Claxton, Maureen, Cropper PH-VE, David de Ferranti, Anil B. Deolalikar, Frederico Guanais,, Dean T. Jamison SK, Jeremy A. Lauer, Lucy O'Keeffe, Damian Walker, Dale, Whittington TW, David Wilson, and Brad Wong. Reference Case Guidelines for Benefit-Cost Analysis in Global Health and Development.
7. Pitt C, Goodman C, Hanson K. Economic Evaluation in Global Perspective: A Bibliometric Analysis of the Recent Literature. *Health Econ* 2016; **25 Suppl 1**(Suppl Suppl 1): 9-28.
8. Sim SY, Jit M, Constenla D, Peters DH, Hutubessy RCW. A Scoping Review of Investment Cases for Vaccines and Immunization Programs. *Value in Health* 2019; **22**(8): 942-52.
9. Husereau D, Drummond M, Augustovski F, et al. Consolidated Health Economic Evaluation Reporting Standards 2022 (CHEERS 2022) statement: updated reporting guidance for health economic evaluations. *BMJ* 2022; **376**: e067975.
